# Modulating serine palmitoyltransferase‐deoxysphingolipid axis in cancer therapy

**DOI:** 10.1002/mco2.44

**Published:** 2020-12-31

**Authors:** Yuancai Xiang, Kun Zhao, Yi‐Quan Tang, Rongyang Dai, Hongming Miao

**Affiliations:** ^1^ Department of Biochemistry and Molecular Biology Third Military Medical University (Army Medical University) Chongqing China; ^2^ Department of Biochemistry and Molecular Biology Southwest Medical University Luzhou China; ^3^ MRC Laboratory of Molecular Biology Cambridge Biomedical Campus Cambridge UK

## Abstract

A schematic illustration is given regarding serine restriction on tumor growth. Once the cellular abundance of serine decreased or alanine accumulated, the serine palmitoyltransferase (SPT) alternatively conjugates alanine and palmitoyl‐CoA to form 3‐keto‐intermediates, which is rapidly converted to 1‐deoxysphinganine and further metabolized to 1‐deoxydihydroceramide (1‐DeoxyDHCER) and 1‐deoxyceramide (1‐DeoxyDHCER), so that to exert cytotoxicity for tumor suppression.

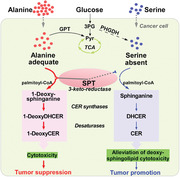

A recent article published in *Nature* by Muthusamy and colleagues demonstrates that the growth of anchorage‐dependent and ‐independent cells possesses different preferences on serine utilization, and that serine restriction increases serine palmitoyltransferase (SPT)‐mediated deoxysphingolipid synthesis, resulting in tumor suppression.^1^ Adopting multiple methods based on SPT promiscuity to regulate the cellular deoxysphingolipids can manipulate the anchorage‐independent growth of cancer cells, which offers us a new insight for tumor treatment.

Metabolic reprogramming has been extensively elucidated in tumor cells since Warburg effect was discovered in 1920s. Now, metabolic reprogramming is a typical hallmark of tumor cells including glucose, fatty acids, and amino acids metabolism. All of them are essential for maintaining the quick demands of substrates and energy in cell proliferation. It is well documented that amino acids such as glutamine, serine, and glycine serve as substrates for new protein synthesis and function as metabolites or metabolic regulators in cancer cell growth. Naturally, owing to the excessive uptake of nutrients, cancer cells are located in a nutrient‐deficient (including amino acids) microenvironment, and indeed, it is reported that restraining the synthesis of certain amino acids can inhibit tumor progression in in vitro and in vivo models.^2^ However, the detailed mechanisms by which amino acids modulate the vitality of tumor and normal cells remain largely unclear. Therefore, identification and distinction of the specific signals related to amino acids metabolism in tumors and hereby developing their targeted therapies will be promising strategies for cancer treatment. Recently, Muthusamy and colleagues unmasked a novel mechanism by which serine restriction constrained tumor growth (Figure 1).

**FIGURE 1:**
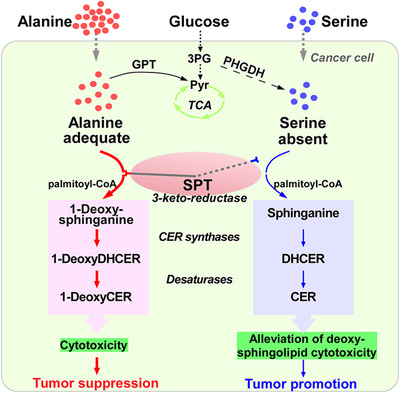
A schematic illustration of serine restriction on tumor growth. Once the cellular abundance of serine decreased or alanine accumulated, the serine palmitoyltransferase (SPT) alternatively conjugates alanine and palmitoyl‐CoA to form 3‐keto‐intermediates, which is rapidly converted to 1‐deoxysphinganine and further metabolized to 1‐deoxydihydroceramide (1‐DeoxyDHCER) and 1‐deoxyceramide (1‐DeoxyDHCER), so that to exert cytotoxicity for tumor suppression. On the contrary, SPT prefers binding to adequate serine to decrease cellular deoxysphingolipids level for alleviating its cytotoxity and maintaining cell growth. Additionally, mitochondrial pyruvate carrier inhibition can promote alanine to convert to pyruvate (Pyr) for energy production, resulting in deoxysphingolipids reduction, thus supporting cell growth. 3PG, 3‐phospho‐glycerate; CER, ceranine; GPT, glutamic pyruvic transaminase; PHGDH, phosphoglycerate dehydrogenase; TCA, tricarboxylic acid

In this study, Muthusamy et al. analyzed the discrepancy of amino acid metabolites between adherent cultured and spheroid cultured cells (anchorage‐independent cell growth model) by using gas chromatography–mass spectrometry. They observed that alanine secretion was markedly increased in spheroid cultured cancer cells, accompanied by elevated pyruvate dehydrogenase phosphorylation. In order to validate the relationship between mitochondrial metabolism and alanine levels, they inhibited pyruvate transport by targeting mitochondrial pyruvate carrier (MPC) and found that alanine secretion was greatly reduced in both culture types of cell, whereas only spheroid cell growth was enhanced significantly. Subsequently, metabolic tracing studies on glucose and alanine showed reductions in MPC potentiated alanine oxidation and serine synthesis. In addition, alanine supplement decreased spheroid biomass and alleviated the propulsive effect of MPC inhibition on spheroid growth. Interestingly, the limited spheroid growth induced by alanine was also caused by serine and glycine deprivation, indicating that a complex amino acids transformation metabolism context could regulate the anchorage‐independent growth of cancer cells.

To unravel the underlying mechanism by which the serine and alanine related‐metabolism modulates spheroid growth, the authors compared the *K*_M_ values of serine‐consuming enzymes in subcutaneous xenograft tumors from the mice fed with serine‐ and glycinv‐free (‐SG) diets or normal diets. These experiments delineated that the only ubiquitously expressed enzyme serine palmitoyltransferase (SPT) exhibited low affinity for serine. Typically, SPT conjugates serine and palmitoyl‐CoA to form 3‐keto‐sphinganine, which is rapidly converted to sphinganine and further metabolized to dihydroceramide, ceramide, and other related complex.^3^ Moreover, SPT displays promiscuous activity that can catalyze multiple amino acids to form different products. For example, SPT concatenates alanine and palmitoyl‐CoA to yield 1‐deoxysphinganine (m18:0) (deoxysphinganine), which is gradually metabolized to other deoxysphingolipids like 1‐deoxydihydroceramide (DeoxyDHCER) and 1‐deoxyceramide (DeoxyCER) through the enzyme system in sphinganine metabolism.^3^ In order to determine whether the diversity of deoxysphingolipids is altered in ‐SG cultured cancer cells, Muthusamy et al quantified the diversity of sphingoid bases using liquid chromatograph–tandem mass spectrometry, revealing that deoxysphinganine was significantly accumulated in serine restriction (or alanine supplementation) and induced spheroid growth inhibition, which was consistent with the outcomes in MPC knockdown. Furthermore, exogenous deoxysphinganine blocked the ability of MPC inhibition to enhance spheroid growth (Figure 1). These data indicated that accumulation of deoxysphinganine might be the main cause resulting in inhibition of spheroid cells’ growth. However, it is well documented that deoxysphinganine becomes toxic upon N‐acylation and formation of DeoxyDHCER.^4^ Consequently, they inhibited deoxysphinganine metabolic enzyme, ceramide synthase, with fumonisin B1 to examine whether deoxysphinganine possessed the function of tumor suppression. As expected, spheroid growth was enhanced under the treatment of fumonisin B1. Furthermore, ceramide species analysis showed that DeoxyDHCER rather than DeoxyCER is markedly increased in ‐SG medium cultured cells (tumor suppression) and decreased in MPC inhibitor setting (tumor promotion). More importantly, the deoxysphinganine and deoxysphingolipids (e.g., DeoxyDHCER and DeoxyCER) were the most upregulated types of sphingoid base in tumor tissues from mice fed with ‐SG diets, but their accumulation only showed a modest upregulation in other tissues such as liver. Collectively, those results suggested that tumor cells possessed a greater proclivity to synthesize or accumulate cytotoxic deoxysphingolipids than other tissues during the restriction of serine and glycine, which represented a potentially effective way for cancer treatment.

It is worth to note that supplementing the potency of isonitrogenous in ‐SG diets by elevating alanine had no additional effect on tumor alanine abundance and tumor growth, indicating that deoxysphingolipids accumulation resulted from serine restriction, which was radically modulated by SPT and was the driver of tumor suppression. In addition, the authors rescued the growth of xenografts tumor in mice on ‐SG diets through inhibiting SPT and deoxysphingolipid synthesis. And unsurprisingly, tumor growth was pronouncedly restricted, as accompanied by deoxysphingolipids accumulation, when blocking the de novo synthesis of serine with PHGDH inhibitor.

In summary, the work by Muthusamy et al. elucidated that serine restriction impeded tumor growth via promoting SPT‐mediated cytotoxic deoxysphingolipids accumulation (Figure 1), which sheds light on amino acids metabolism‐based strategies for cancer therapy. This simple and effective method makes serine restriction strategy own a broad prospect for clinical application. Of note, the inhibitory effect of tumor growth triggered by serine restriction is a complex process that may involve multiple aspects such as enhancing mitochondrial fragmentation by regulating lipid metabolism,^5^ or alleviating nucleotide synthesis through limiting one‐carbon units.^6^ Additionally, a phenotype of macular telangiectasia type 2 and peripheral neuropathy may occur when the level of serine in patients is low for a long time,^7^ elevating these potential risks of serine restriction treatment. Moreover, a neurotoxicity of 1‐deoxysphingolipids raised by the expression of SPT variants, *SPTLC1* and *SPTLC2*, also could account for rare autosomal dominant disorder, hereditary sensory and autonomic neuropathy type 1 (HSAN1).^8^ Thus, it is necessary to decode the mechanisms of how the molecular switch SPT selectively binds to different amino acid substrates and how these deoxysphingolipids exert cytotoxicity in the future work. An understanding of these information can help us develop other deoxysphingolipids‐based therapies to avoid macular telangiectasia type 2, peripheral neuropathy, and HSAN1, thus providing personalized treatments.

## CONFLICT OF INTEREST

The authors declare no conflict of interest.

## AUTHOR CONTRIBUTIONS

X.Y. and M.H. discussed and wrote the manuscript. Z.K., T.Y., D.R., and M.H. revised this manuscript.
